# Clinical analysis of 33 cases with neonatal cerebral infarction

**DOI:** 10.12669/pjms.37.7.4720

**Published:** 2021

**Authors:** Ning Yang, Xiaojun He, Cuixia Yin, Lihua Zhao

**Affiliations:** 1 Ning Yang, Neonatal Department, Dezhou People’s Hospital, Dezhou 253000, Shandong, China; 2 Xiaojun He, Neonate Department, Ningjin County People’s Hospital, Dezhou 253400, Shandong, China; 3 Cuixia Yin, Neonate Department, Ningjin County People’s Hospital, Dezhou 253400, Shandong, China; 4 Lihua Zhao, Neonate Department, Ningjin County People’s Hospital, Dezhou 253400, Shandong, China

**Keywords:** Cerebral Infarction, Clinical Analysis, Infant, Newborn

## Abstract

**Objective:**

To investigate the etiology, clinical manifestations, diagnosis, treatment and prognosis of neonatal cerebral infarction (NCI) to further improve the understanding of the disease.

**Methods:**

Clinical data and follow-up results of 33 cases of NCI in neonatal intensive care unit of a first-class hospital from September 2009 to September 2019 were retrospectively analyzed.

**Results:**

All 33 patients were diagnosed with NCI by MRI. Among them, 31 cases (93.94%) were full-term infants, 25 cases (75.76%) were mother’s first birth, and 18 (54.55%) cases were males. Pregnancy complications were reported in 18 cases (54.55%), and 19 cases (57.58%) had perinatal hypoxia history. Seizures were the most common first symptom and clinical manifestation in the course of disease (81.8%). There were 27 cases (81.82%) of patent foramen ovale (PFO) among NCI cohort. Ischemic cerebral infarction occurred in 32 cases (96.97%). The middle cerebral artery and its branches were more frequently involved, mainly on the left side. The acute stage of NCI was managed by symptomatic support treatment, and the recovery stage involved mainly rehabilitation treatment. Among the 33 cases, five cases were lost to follow-up, two patients died, 26 patients survived without complications, one case had cerebral palsy, one case had language retardation, and six cases had dyskinesia. Poor prognosis was associated with the involvement of deep gray matter nuclei or multiple lobes, and intrapartum complications. Vaginal mode of delivery and longer hospital stay were associated with better prognosis.

**Conclusions:**

Complications leading to placental circulation disorder during pregnancy and perinatal hypoxia are common high-risk factors of NCI. The seizure is the most common clinical manifestation. There is a possible correlation between PFO and NCI. Involvement of deep gray matter or multiple lobes and intrapartum complications may indicate poor prognosis, while vaginal delivery and prolonged hospitalizations are associated with better prognosis of NCI.

## INTRODUCTION

Neonatal cerebral infarction (NCI), also known as neonatal cerebral stroke, refers to the occlusion of one or more branches of neonatal cerebral vessels within 28 days after birth due to various reasons, leading to focal or multifocal ischemic necrosis in the corresponding blood supply area.^1^ Cerebral infarction includes arterial ischemic stroke (AIS) and hemorrhagic infarction. AIS is the most common type of cerebral infarction and accounts for about 90% of all the NCI cases.^2^ It can occur in the anterior, middle and posterior cerebral arteries, with the left middle cerebral artery being the most common location.^3^ At present, the risk factors of NCI are still unclear, and the clinical manifestations are diverse, making early diagnosis difficult. Recent developments in neuroimaging technology in newborns has allowed to establish that NCI is not uncommon, with the incidence rate of about 1 / 1600-3000,^1^ markedly higher than the incidence of cerebral infarction in children and similar to the rate in elderly adults.^4^,^5^ In some cases, NCI may leave some sequelae such as dyskinesia, cognitive impairment, cerebral palsy and epilepsy that have a significant negative impact on the quality of life as well as financial burden to the healthcare system.^6^-^8^ In the recent study, the clinical data and prognosis of 33 cases of NCI in a first class hospital were retrospectively analyzed to provide clinical research data for further understanding the characteristics of NCI.

## METHODS

Medical records of children with NCI, 18 males (54.55%) and 15 females (45.45%), were retrospectively analyzed in the Department of Neonatology of a first-class hospital from September 2009 to September 2019. This study was approved by the medical ethics committee of Dezhou People’s Hospital (Ref. 20210079, date of approval: 2021 April 09^th^). Informed consent was obtained from the guardians of the children.

Clinical data were collected and recorded, including gestational age, gender, birth weight, maternal disease history, family history, perinatal hypoxia history, perinatal infection history, delivery situation, cardiac color Doppler ultrasound examination, blood routine examination, coagulation function, first symptom, hospitalization time, short-term and long-term prognosis, magnetic resonance imaging (MRI) examination results.

For newborns with clinical history of intrauterine distress or asphyxia during labor and clinical manifestations of convulsion, poor reaction, cyanosis, recurrent apnea and severe jaundice, the head MRI scan was performed, including transverse T1 weighted imaging (T1WI), T2 weighted imaging (T2WI), T2 fluid attenuated inversion recovery (T2-FLAIR), diffusion weighted imaging (DWI) and sagittal T1WI. In cases of NCI, parallel Magnetic resonance angiography (MRA) was performed based on the results of MRI.

### Statistical Analysis

Statistical analysis was performed using R Software Version 3.5.3 (R Core Team, 2019). Categorical variables were presented as counts and % of total and comparisons based on prognosis were made using chi-square tests. Distribution of continuous data was determined using the Kolmogorov-Smirnov test. Continuous variables were presented as mean and ± standard deviation (SD) and median and interquartile range (IQR). Comparisons based on prognosis for non-parametric variables were made using Mann-Whitney U tests. For all tests, p-values < 0.05 were considered statistically significant. Prognosis was determined by medical assessment made during follow-up visits starting at two weeks after discharge and continuing once a month until the patient reaches two years of age.

## RESULTS

As summarized in Table-I, the mean gestational age, mean(±SD), of 33 hospitalized NCI infants included in the study was 38.52 (±1.42) weeks. The average maternal age was 28.79 (±3.52) years and mean paternal age was 29.8 (±5.3) years. All the included cases were single births, and 25 (75.76%) were first births. There were 17 cases of cesarean section (51.52%) and 16 cases of vaginal delivery (48.48%). Two infants were premature (6.06%) and 31 were full-term (93.94%). The average birth weight was 3.37(±0.54) kg, including one low birth weight infant (3.03%) and four macrosomia infants (12.0%). Seven percent of infants had Apgar score of 10 at one minute, rising to 26% at five minutes after birth. The earliest NCI onset was postnatal, and the latest onset was 20 days after birth. The age of admission ranged between one hour and 20d after birth. Among them, 26 patients (78.79%) were admitted within three days after birth, with an average length of stay of15.45 (±13.61) days (Table-I).

**Table I:** Demographic and delivery variables (N=33).

		*Cases (%)*	*Mean (±SD)*
Gender	Male	18 (54.5)	
	Female	15 (45.5)	
Gestational age (weeks)	Premature (<37)	2 (6.1)	38.5 (1.4)
	Full term (≥37)	31 (93.9)	
Birth weight (g)	Low (<2500)	1 (3.0)	3371.1 (535.4)
	Normal	28 (84.8)	
	High (>4000)	4 (12.0)	
Mother’s age (years)	≥35 years	2 (6.1)	28.8 (3.5)
	<35 years	31 (93.9)	
Father’s age (years)	≥35 years	7 (21.2)	29.8 (5.3)
	<35 years	26 (78.8)	
Delivery method	Vaginal	16 (48.5)	
	Caesarean	17 (51.5)	
Gravidity	Primigravida	18 (54.5)	
	Multigravida	15 (45.5)	
Parity	Primipara	25 (75.8)	
	Multipara	8 (24.2)	
Apgar score at 1 min	10	7 (21.2)	
	9 or less	26 (78.8)	
Apgar score at 5 min	10	27 (81.8)	
	9 or less	6 (18.8)	
Prenatal complications	Present	18 (54.5)	
	None	15 (45.5)	
Intrapartum complications	Present	14 (42.4)	
	None	19 (57.6)	
Prognosis	Normal	18 (54.5)	
	Adverse outcomes	10 (30.3)	
	Loss to follow-up	5 (15.2)	
	Mean (±SD)	Median (IQR)	
Age at onset (hours)	50.6 (89.4)	21 (4-48)	
Length of stay (days)	12.2 (13.9)	9 (7-14)	

All categorical variables displayed as cases and % of total. All continuous variables displayed as mean ±SD, as cases (%) among categories of relevance to birth outcomes, or as mean ±SD and median (IQR). Prematurity was defined as <37 weeks gestational age, low birth weight was defined as <2500g birth weight, and advanced parental age was defined as ≥35 years. Prenatal complications include diabetes, anemia, subclinical hyperthyroidism, prenatal fever, hypothyroidism, and vaginitis. Intrapartum complications include amniotic fluid meconium, PROM, oligohydramnios, nuchal cord, and resuscitation.

In 54.5% of included cases there was a normal prognosis of NCI, 30.3% reported adverse outcomes, and 15.2% of the cases were not followed-up. There were 18 recorded cases (54.55%) of gestational complications, which included gestational diabetes mellitus, hypothyroidism, subclinical hyperthyroidism, antenatal cold and fever, anemia during pregnancy, and vaginitis. Intrapartum complications, such as amniotic fluid meconium, premature rupture of membranes, oligohydramnios, nuchal cord, and resuscitation were reported in 42.4% of the cases.

### Clinical manifestations and complications

Clinical manifestations and complications of NCI patients are summarized in Table-II. Among the 33 cases, 25 (75.76%) were admitted to hospital with seizures before admission as the major complaint, five cases were admitted with a weak reaction, two cases with jaundice and one case with pale skin. The average onset time of seizures was 44.8 hours after birth. Two patients were admitted to hospital for skin jaundice without any neurological manifestations. EEG results were abnormal in 72.7% of the examined. Comorbidities of the 33 NCI patients were as follows: five had purulent meningitis, three had neonatal sepsis, two had atrial septal defect, 27 had patent foramen ovale, three had patent ductus arteriosus and one had decreased left ventricular systolic function. Out of 33 cases, 30 NCI patients improved and were discharged, and three patients were discharged automatically after considering the prognosis. Five cases were lost the follow-up due to interruption or refusal of contact information (Table-II). After admission, 33 cases were given etiologic and symptomatic support treatment. Patients with seizures were given phenobarbital sodium to control the convulsions.

**Table II:** Clinical characteristics (N=33).

		*Cases (%)*
Clinical manifestation of NCI	Seizure	25 (75.8)
	Weak reaction	5 (15.2)
	Jaundice	2 (6.1)
	Pale	1 (3.0)
EEG results	Abnormal	24 (72.7)
	Normal	3 (9.1)
	Not performed	6 (18.2)
Patent foramen ovale	Present	27 (81.8)
	Absent	4 (12.1)
	Test not performed	2 (6.1)

All categorical variables displayed as cases and % of total.

There were 27 cases (81.82%) with convulsions before or after admission (Table-III). There were 13 cases with unilateral limb twitch, 10 cases on the right side, and three cases on the left side. There were four cases with paroxysmal cyanosis, apnea, limb shaking, and one case was binocular gaze.

**Table III:** Characteristics of the reported seizures (N=27).

		*Cases (%)*
Clinical manifestation of seizures	Unilateral limb twitch	13 (48.14)
	Right limb twitch	10 (37.1)
	Left limb twitch	3 (11.1)
	Paroxysmal cyanosis	4 (14.8)
	Binocular gaze	1(3.7)

All categorical variables displayed as cases and % of total.

### Auxiliary examination

**Fig.1:**
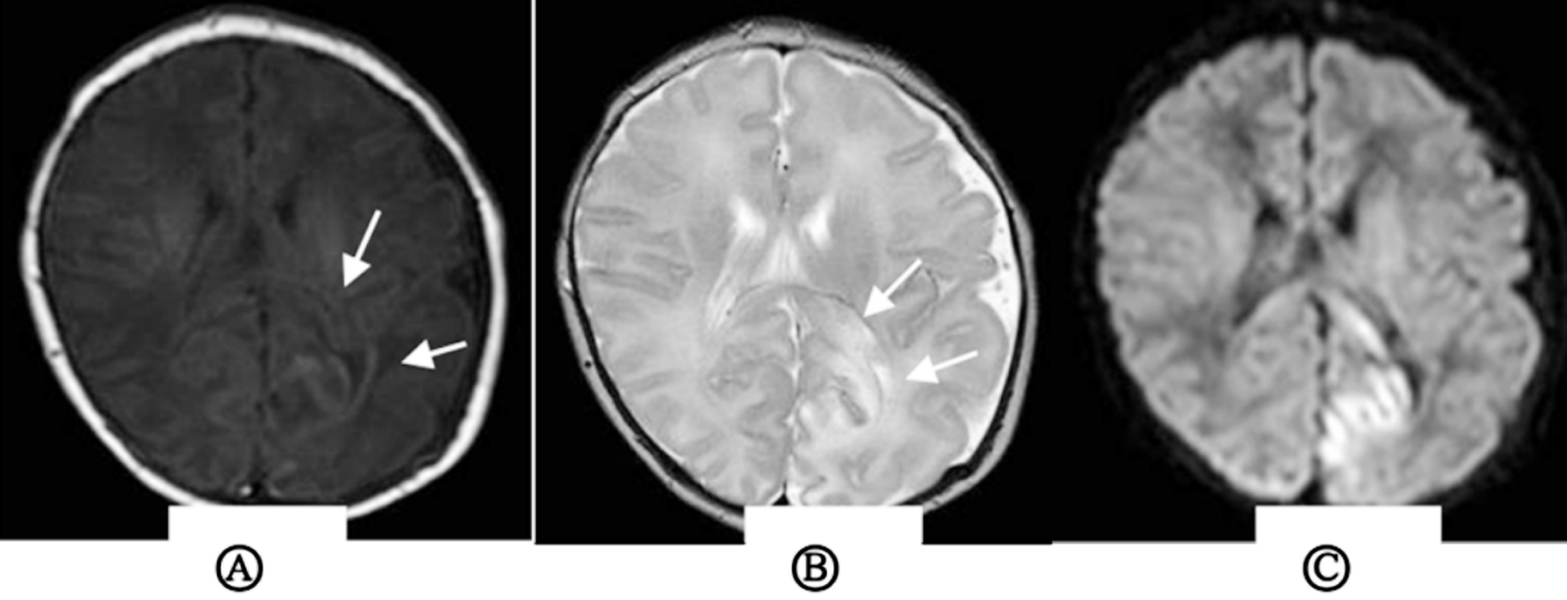
Magnetic resonance imaging findings in the left occipital of neonates with cerebral infarction A) as indicated by the arrows, T1WI suggests a large area of long T1 signal in the left occipital; B) as indicated by the arrow, T2WI suggests a large area of long T2 signal in the lesion area; C) diffusion weighted imaging (DWI) indicates a significant signal in the lesion.

All 33 cases were examined by plain MRI that showed patchy T1 and T2 signals in the infarction area, and high signals in the infarction area on DWI (Fig.1). The lesions were located in the right side in 11 cases, the posterior cerebral artery in 3 cases, the anterior cerebral artery in one case, and the middle cerebral artery in seven cases; the lesions were located in the left side in 21 cases, the posterior cerebral artery in one case, and the middle cerebral artery in 20 cases; one case had a hemorrhage. Twenty-nine patients underwent MRA examination. The lumen of the feeding artery was narrowed in 18 cases, and the compensatory thickening of feeding artery was found in six cases (82.76%). No abnormality was found in five cases.

**Fig.2:**
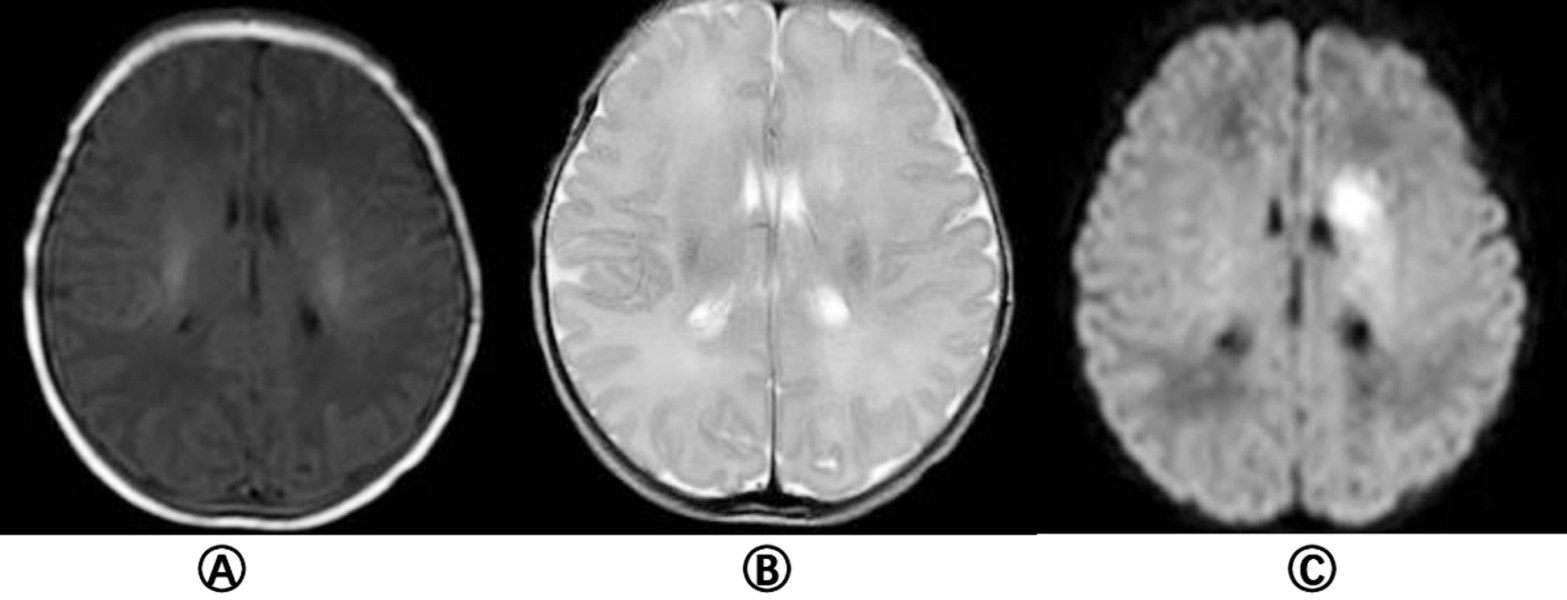
MRI findings of left basal ganglia in neonatal cerebral i nfarction a) T1WI showed long T1 signal in the left basal ganglia; B) T2WI showed long T2 signal in the lesion area; C) Diffusion weighted imaging (DWI) showed patchy hyperintensity in the left basal ganglia.

Twenty-six patients received ambulatory electroencephalogram (AEEG) examination during their hospitalization. Three cases had normal AEEG results, and 23 were abnormal, presenting as sharp wave, spike wave, slow wave, spike slow wave, sharp slow wave and low voltage during sleep, etc.

We next evaluated the possible effect of demographic parameters and delivery method on the prognosis of NCI patients. As shown in Table-IV, gender, gravidity, parity and lower Apgar score at five minutes did not impact the prognosis (P>0.05). On the other hand, vaginal birth was associated with a better prognosis, as compared to caesarian section (P=0.037).

**Table IV:** Comparison of prognosis among demographic and delivery variables.

		*Normal Cases (%)*	*Adverse Outcomes Cases (%)*	*p-value*
Gender	Male	10 (58.8)	7 (41.2)	0.453
	Female	8 (72.7)	3 (9.1)	
Delivery method	Vaginal	11 (84.6)	2 (15.4)	*0.037
	Caesarean	7 (46.7)	8 (53.3)	
Gravidity	Primigravida	11 (73.3)	4 (26.7)	0.283
	Multigravida	7 (53.8)	6 (46.2)	
Parity	Primipara	14 (70.0)	6 (30.0)	0.318
	Multipara	4 (50.0)	4 (50.0)	
Apgar score at 5 min	10	16 (69.6)	7 (30.4)	0.211
	9 or less	2 (40.0)	3 (60.0)	

All variables were compared between cases with a normal prognosis (N = 18) and cases with adverse outcomes (N = 10) and excluded loss to follow-up cases (N = 5). Adverse outcomes included motor dysfunction, speech or motor delay, limb weakness or inflexibility, appendage adduction, and death. Comparisons were made using a chi-square test, with p < 0.05

* considered statistically significant.

**Table V:** Comparison of prognosis among clinical characteristics.

		*Normal Cases (%)*	*Adverse Outcomes Cases (%)*	*p-value*
Prenatal complications	Present	9 (52.9)	8 (47.1)	0.119
	None	9 (81.8)	2 (18.2)	
Intrapartum complications	Present	4 (40.0)	6 (60.0)	*0.046
	None	14 (77.8)	4 (22.2)	
Clinical manifestation of NCI	Seizure	14 (63.6)	8 (36.4)	0.891
	Other	4 (66.7)	2 (33.3)	
Patent foramenovale	Present	16 (72.7)	6 (27.3)	0.365
	Absent	2 (50.0)	2 (50.0)	
		Median (IQR)	Median (IQR)	p-value
Age at onset (hours)†		22.5 (13-48)	16 (4.5-48)	0.441
Length of stay (days)†		9.5 (8-15)	7 (4-9)	*0.041

All variables were compared between cases with a normal prognosis (N = 18) and cases with adverse outcomes (N = 10) and excluded loss to follow-up cases (N = 5). Adverse outcomes included motor dysfunction, speech or motor delay, limb weakness or inflexibility, appendage adduction, and death. Comparisons were made using a chi-square test for categorical variables or a Mann-Whitney U-test for non-parametric continuous variables†, with p < 0.05

* considered statistically significant.

Prenatal complications, clinical manifestations of NCI, presence of foramen ovale and the age of NCI onset did not affect the outcome (P>0.05). However, intrapartum complications were associated with worse prognosis (P=0.046), and longer hospital stay correlated with better prognosis of NCI patients (P>0.041).

## DISCUSSION

The development of imaging technology in recent years significantly improved the diagnosis rate of neonatal cerebral infarction. Nevertheless, the etiology and pathogenesis of NCI are still not completely clear, and there is a great need for effective early clinical interventions. NCI can be divided into ischemic cerebral infarction and hemorrhagic cerebral infarction, with arterial ischemic infarction (AIS) accounting for the majority of the lesions.^9^ In this study, 96.9% of the cases (32 out of 33) were diagnosed as AIS, a higher rate than that reported in the literature, probably due to the overall small number of cases. Previous studies have shown that the incidence of NCI in male is high, and the ratio of male to female is 1.3-1.6:1.^10^-^12^ Our results are in agreement with these reports, with males accounting for 54.55% of all patients.

The etiology of NCI is complex, with multiple contributing factors. Studies have shown that conditions, such as chorioamnionitis during pregnancy, preeclampsia, diabetes, autoimmune system related diseases, thrombotic diseases, infertility, primipara, smoking, fever, fetal heart rate decline during delivery, amniotic fluid and meconium contamination, emergency cesarean section, one minute Apgar score < 3 points, and five minutes Apgar score < 7 points all contribute to higher risk of developing NCI.^13^-^16^ In this study, there were 18 cases of pregnancy complications reported, including gestational diabetes mellitus, thyroid dysfunction, and infections. These conditions may potentially result in abnormal endothelial cell coagulation activity, uterine blood perfusion reduction, placental circulation disorder and dysfunction, as well as cerebrovascular regulation disorders, cerebral vasospasm, eventually leading to fetal and neonatal cerebral vasospasm infarction. Some studies have confirmed that NCI is more common in primiparas, probably because primipara increases the incidence of delivery complications, which can lead to asymptomatic hypoxia and ischemia in fetus or newborn. Although these conditions are not enough to cause hypoxic-ischemic encephalopathy, they may cause NCI in newborns with AIS.^10^ Our data show that 25 cases (75.76%) were firstborn, much higher than the percentage of non-primiparas (24.24%), further confirming these reports.

Previous studies suggest that factors, such as vacuum delivery and emergency Caesarian section, are associated with an increased risk of NCI.^17^,^18^ In agreement with these reports, we show that vaginal delivery in our study is associated with an overall better prognosis of NCI as compared to caesarian section. Our study also showed that intrapartum complications in children with NCI were associated with a poor prognosis. Interestingly, patients with normal prognosis had significantly longer median length of hospitalization (9.5 days as compared to 7). This finding could indicate that longer hospital stays, and more healthcare resources and rehabilitation delivered to the patient are contributing to the better overall prognosis of NCI.

Severe infections and complications, such as sepsis and meningitis, are also important causes of cerebral infarction. NCI, secondary to bacterial meningitis, is often more severe and has worse prognosis.^19^ In this study, 5 cases of children with purulent meningitis were reported, but the prognosis was favorable. The relationship between these high-risk factors and AIS remains to be further confirmed.

In recent years, the link between patent foramen ovale (PFO) and cerebral infarction has become a research hotspot. In adults, PFO is considered an important risk factor for ischemic stroke.^20^ When the right atrial pressure increases, thrombosis in the venous system or cardiac cavity can enter the left ventricular system through the patent foramen ovale, resulting in cerebral embolism.^21^ However, foramen ovale is a normal blood circulation channel during the fetal period. After birth, the normal pulmonary circulation is established and left atrial pressure is increased, leading to gradual closure of foramen ovale. The unclosed foramen ovale in newborns is, therefore, temporal and physiological, and most of them are gradually closed within one year after birth. Studies have shown that 62% of full-term, healthy newborns have PFO in the first 60 hours after birth. 22 In this group, 27 cases (81.82%) had PFO, all of which were small and medium-sized. The average diameter of foramen ovale was (2.63 ± 0.51) mm. We may hypothesize that the cause of NCI in these cases is due to the increase of pulmonary artery pressure, which leads to the increase of right atrial pressure. After the embolus of the venous system returns to the right heart through the vena cava, it enters the left heart directly through the patent foramen ovale, and then flows into the systemic circulation, reaching and blocking the intracranial artery, which leads to cerebral ischemia. More studies are merited to establish the exact correlation between NCI and PFO.

Seizure is the most common clinical manifestation of NCI that occurs in 70% to 90% of children with NCI.^22^ Seizures usually occur 12-72 hours of birth^2^ and are usually manifested as local convulsions on the opposite side of the lesion. Our study shows that seizure is still the most common first symptom and the most common clinical manifestation of NCI. 75.76% of the children in our report were admitted to the hospital with convulsion as the main complaint, and 81.82% of the children had convulsions during the course of the disease. The average time of seizure was about 48 hours after birth, and about half of the children presented with contralateral limb twitching, which was consistent with the literature reports.^2^ Most of the children’s clinical symptoms appear within three days after birth. Therefore, we should be alert to cerebral infarction, especially in children with unilateral limb twitch. In addition, other non-specific manifestations, such as paroxysmal cyanosis, apnea, poor reaction, severe jaundice, anemia of unknown causes, should all be evaluated to exclude intracranial lesions.

Cranial magnetic resonance imaging (MRI), including conventional MRI, diffusion weighted imaging (DWI) and magnetic resonance angiography (MRA), is the golden standard for NCI diagnosis.^23^ Conventional MRI of NCI brain usually shows T1WI low signal and T2WI high signal change within one week after the onset of NCI, and high signal on T1WI and low signal on T2WI in lesion area one week later. DWI shows high signal in lesion area several hours after NCI onset, and the lesion location displayed in that early stage has a certain correlation with the clinical prognosis.^23^ Studies have reported that when DWI indicates early-stage damage to the internal capsule, basal ganglia, cerebral peduncle and other parts, it correlates with the increased incidence of long-term dyskinesia in children.^24^ MRA is a noninvasive vascular imaging reconstruction technique, that does not require contrast agent, and is utilized to diagnose cerebrovascular diseases, cerebrovascular stenosis, occlusion or malformation.^25^ In this study, 28 cases of acute or subacute cerebral infarction showed as isointense or slightly hypointense on T1WI, isointense or slightly hyperintense on T2WI and T2-FLAIR, hyperintense on DWI, and exhibited decreased apparent diffusion coefficient (ADC). Five cases of chronic cerebral infarction showed hyperintensity on T1WI, low signal on T2WI and T2-FLAIR, isointense or hypointense on DWI. Our study showed that the middle cerebral artery was most frequently involved in NCI, and the left side was higher than the right side.

While MRI combined with MRA is helpful for early detection of vascular abnormalities, studies have shown that early EEG has certain guiding significance for the evaluation of brain function and prognosis. EEG has been extensively used in children with seizures, suspected convulsions, and subclinical convulsions without typical clinical manifestations.^26^ In this study, 26 cases were examined by AEEG. Of them, abnormalities were detected in 23 cases, mainly manifested as abnormal high amplitude discharge and slow electrical activity. However, abnormal EEG had little correlation with poor prognosis. Further larger long-term follow-up studies are needed to evaluate the prognostic value of abnormal EEG in children with NCI.

The treatment of NCI is mainly symptomatic and supportive in the acute stage to maintain the stability of internal environment. Phenobarbital is used to stop spasms in patients with seizures, and rehabilitation treatment is mainly used in the recovery period. The reports on prognosis of neonatal cerebral infarction are quite different,^4^-^6^ and depend on the evaluation method, type and scope of infarction, and follow-up time. However, neonatal cerebral infarction is still one of the main causes of motor disorders, cerebral palsy, epilepsy, audiovisual and cognitive impairment.^27^ The incidence of sequelae in our study was 35.7% (10/28). The extent of cerebral infarction in children with poor prognosis mostly involved deep gray matter nuclei and multiple lobes, which was consistent with the literature reports.^6^

### Limitation of the study

It includes small sample size. Although certain factors, including low birth weight, prematurity and low one minute Apgar score seem to indicate possible associations with poorer prognosis, the significance of these factors couldn’t be calculated because of low case counts. Further larger-scale retrospective studies are needed to evaluate clinical impact of these factors on the prognosis of NCI.

## CONCLUSION

Neonatal cerebral infarction is one of the brain injuries that seriously threaten the prognosis of the neonatal nervous system. Perinatal hypoxia is the main cause of cerebral infarction. Convulsion is the most common early clinical manifestation. Early detection and diagnosis combined with cranial imaging examination are of positive significance for judging prognosis, guiding rehabilitation treatment and improving prognosis.

### Authors’ Contributions:

**NY and XH** conceived and designed the study. CY and LZ collected the data and performed the analysis. **NY and XH** were involved in the writing of the manuscript and the integrity of the study. **NY** edited the manuscript.**NY and XH** are responsible for integrity of the study. All authors have read and approved the final manuscript.
